# Improved heterogeneity handling in the collapsed cone dose engine for brachytherapy

**DOI:** 10.1002/mp.17434

**Published:** 2024-10-29

**Authors:** Freja Alpsten, Bob van Veelen, Christian Valdes‐Cortez, Francisco Berumen, Anders Ahnesjö, Åsa Carlsson Tedgren

**Affiliations:** ^1^ Department of Oncology‐Pathology Karolinska Institutet Stockholm Sweden; ^2^ Department of Nuclear Medicin and Medical Physics Karolinska University Hospital Stockholm Sweden; ^3^ Elekta Brachytherapy Veenendaal The Netherlands; ^4^ Nuclear Medicine Department Hospital Regional de Antofagasta Antofagasta Chile; ^5^ Service de Physique Médicale et de Radioprotection Centre Intégré de Cancérologie CHU de Québec – Université Laval et Centre de recherche du CHU de Québec Quebec Canada; ^6^ Département de Physique de Génie Physique et d'Optique et Centre de Recherche sur le Cancer Université Laval Quebec Canada; ^7^ Department of Immunology Genetics and Pathology Uppsala University Uppsala Sweden; ^8^ Department of Health Medicine and Caring Sciences Linköping University Linköping Sweden

**Keywords:** brachytherapy, collapsed cone, model‐based dose calculation algorithm

## Abstract

**Background:**

Model‐based dose calculation algorithms (MBDCA), such as the Advanced Collapsed cone Engine (ACE) in Oncentra Brachy® can be used to overcome the limitations of the TG‐43 formalism. ACE is a point kernel superposition algorithm that calculates the total dose separated into primary, first‐scatter, and multiple‐scatter dose. Albeit ACE yields accurate results under most circumstances, several studies have reported underestimations of the dose to cortical bone. These underestimations are likely caused by approximations in the handling of multiple‐scatter dose for non‐water media. Such would result in noticeable deviations where the multiple‐scatter is a considerable fraction of the total dose, that is, at greater distances from the source.

**Purpose:**

To improve and test the accuracy of the multiple‐scatter dose component in the ACE algorithm to remedy its inaccuracy for non‐water geometries.

**Methods:**

A careful analysis of the transport and absorption of the multiple‐scatter energy fluence revealed an inconsistency in the scaling of energy absorption ratios for non‐water media of the multiple‐scatter kernel. We implemented an updated algorithm version, ACE_corr_, and tested it for three different geometries. All had a single ^192^Ir‐source at the center of a cubic water phantom with a box‐shaped heterogeneity of either cortical bone or air, positioned at different distances from the source. Dose distributions for the three cases were calculated with ACE and ACE_corr_ and compared to Monte Carlo simulations, using the percentage dose difference ratio as figure‐of‐merit. All dose calculation methods scored separately the dose deposited by primary, first‐scattered, and multiple‐scattered photons.

**Results:**

The accuracy of the updated algorithm ACE_corr_ was superior to ACE. In the cortical bone heterogeneity, the mean percentage dose difference ratio for the total dose improved from −11.7% to −2.2% (in the worst case) by our update. Less impact was seen in the air heterogeneity, where both ACE and ACE_corr_ deviated less than 2% from the Monte Carlo results. The algorithm update mainly concerns the multiple‐scattered dose component, but an accompanying data processing update also had a small effect (≤0.5% difference) on the primary and first‐scattered dose. The calculation times were not affected.

**Conclusions:**

The updates to ACE improved the accuracy of multiple‐scatter dose calculation for non‐water media, without increasing calculation times.

## 1 INTRODUCTION

The TG‐43 formalism for dose calculations in brachytherapy treatment planning was introduced by Task Group No. 43 (TG‐43) of the American Association of Physics in Medicine (AAPM) in 1995.^1^, ^2^ The formalism became the standard methodology for dose calculations in clinical treatment planning systems (TPS) due to various reasons such as traceability to metrological standards, fast calculation times, and implementation simplicity. However, the TG‐43 formalism suffers limitations as it ignores variations in dose absorption and scatter transport from, for example, patient‐specific tissue distributions, applicator media, and interseed attenuation.^1^, ^2^, ^3^, ^4^ Model‐based dose calculation algorithms (MBDCA) offer a more detailed representation of radiation transport, handling better non‐water media. According to the AAPM TG‐186 report,^3^ three MBDCA algorithms are of interest to brachytherapy: Monte Carlo (MC), deterministic solutions to the linear Boltzmann transport equation, and the collapsed cone kernel superposition method. Although full MC is considered a golden standard for dose calculation accuracy, its long calculation times make it uncommon for clinical applications.^5^

The advanced collapsed cone engine ACE in the Oncentra Brachy® (*Elekta AB, Stockholm, Sweden)* TPS is a collapsed cone point kernel superposition algorithm. ACE utilizes a separation by photon scatter generation method called successive scattering, in which the dose from primary (prim), first‐scattered (1sc), and multiple‐scattered (msc) photons are calculated separately in sequence.^6^, ^7^, ^8^ The dose from a lower scatter order forms the energy release pattern for the subsequent higher scatter order. The primary dose is calculated by simple ray‐tracing from the source, while the 1sc and msc dose distributions are calculated through point kernel superpositions of the correspondingly released energy distributions. This process is based on the collapsed cone method^9^ specifically adapted for brachytherapy.^6^, ^7^, ^8^, ^10^, ^11^, ^12^

Several studies have compared ACE to MC with good results, especially for target volumes.^13^, ^14^, ^15^, ^16^, ^17^, ^18^ Ma et al.^13^ compared ACE to MC for real‐patient cases, including target locations at breast, chest wall, and prostate. They studied the volume receiving ≥100% of the prescribed dose for which they found the maximum deviation of ACE from MC to be less than 3%.^13^ However, several of these studies also report discrepancies between ACE and MC in cortical bone.^13^, ^14^, ^15^, ^16^, ^18^ Dose underestimations in the order of −6% to −15% in cortical bone have been observed in distant low‐dose regions where the multiple‐scatter dose is prominent,^13^, ^14^, ^15^, ^16^, ^18^ see, for example, Fig. 7b in Ma et al.^13^ and Figure 2a in the results section below.

In this work, we verify that the degraded accuracy in cortical bone originates from the msc dose part of the algorithm. We derive a more detailed algorithm including a new scaling factor $\chi_{\mathrm{msc}}$ that better considers the multiple scatter energy transport across media boundaries. We have updated and implemented the algorithm into the amended code ACE_corr_, and present test results in simple single‐source geometries to demonstrate the dosimetric effects of the new approach.

## 2 MATERIALS AND METHODS

The three different dose components; primary, 1sc, and msc, are explicitly modeled in ACE. The primary dose part is fully modeled through straightforward raytracing. The 1sc part has no major limitations apart from the collapsed cone approximation. In this approximation, the fluence and absorption of 1sc photons taking place within discrete cones are collapsed to take place on the respective cone axis. This discretization causes a spiky pattern in the dose deposition originating from a certain site or voxel. However, these spikes are, to a high degree, smeared out since all voxels receiving a primary dose also emit 1sc photons. The approach is also energy‐conservative such that the total energy transferred and absorbed between different generations is correctly quantified. The collapsed cone approximation is also applied for the msc fraction, but the transport model in the original implementation was truncated in such a way that all energy of the second scattered photons was deposited at distances given by the msc kernel exponential parametrization. This approach is still energy‐conservative, but in media where the mass‐energy transfer fraction is larger than for the kernel medium (water), a local underdosage will occur since too little energy is deposited on the spot, and vice versa. The larger the energy absorption differences between media and water, the larger the displacement. Hence, the displacement is most visible for higher atomic number media, for which the photoelectric effect causes larger energy absorption differences versus water at the low energies of the scattered photons.

To remedy the shortcoming, two major changes have been implemented in the msc dose modeling. First, an energy absorption scaling factor for the msc dose component has been introduced (see Section 2.1). Secondly, a data processing update to improve the method for calculating media‐specific interaction coefficients has been designed. In the original implementation, the interaction coefficients for first and multiple scatter calculations were calculated from the photon energy fluence spectrum in a similar manner as described in Section 2.2 in Ahnesjö et al.^7^ For this purpose, the spectrum at one specific depth was chosen, thus, ignoring spectral changes with distance from the source (hardening effect). To account for these effects in ACE_corr_, the interaction coefficients were instead fitted toward values over a distance.

For testing purposes, dose distributions have been calculated with TG‐43, ACE, ACE_corr_, and MC. Except for TG‐43, all total dose distributions *D*_tot_ have been separated into primary dose (*D*_prim_), first‐scatter dose (*D*_1sc_), and multiple‐scatter dose (*D*_msc_). As figure‐of‐merit for comparison the percentage dose difference ratio $\Delta_{\mathrm{gen}}=\;100\;\left(D_{\mathrm{gen}}^{\mathrm{test}}-D_{\mathrm{gen}}^{\mathrm{MC}}\right)/D_{\mathrm{tot}}^{\mathrm{MC}},$ was used, where the subscript “gen” is a placeholder for dose label (prim, 1sc, msc or tot, the latter for the total dose sum of the components), and the superscript “test” can be either ACE or ACE_corr_ with MC indicating Monte Carlo.

### 2.1 Multiple‐scatter algorithm updates

This section builds on the description in Ahnesjö et al.^7^ To simplify, we used identical variables, as summarized in Table 1. The point kernels for both the original and updated algorithms are derived for water and, when used in non‐water media, scaled by interaction coefficient ratios. Forced interaction MC simulations are used for kernel creation and derivation of energy spectra for 1sc and msc photons.^8^ For the msc dose calculation, only an attenuation scaling factor $\eta_{\mathrm{msc}}$ was used in the original implementation, but we now include also an energy absorption scaling factor $\chi_{\mathrm{msc}}$. This factor is defined analogously to the scaling factor used in the 1sc dose calculations (see eq. 44 in Ahnesjö et al.^7^) but specifically for the msc photons, as the ratio of linear energy absorption for a medium “m”, relative to water “w”, that is,

(1) χmsc=ρmρwμ¯en,mscμ¯en,mscρρwm

where the bar indicates averaging over the energy spectrum of multiple‐scattered photons.

**TABLE 1:** List of variables used in algorithm description. The nomenclature is identical to the one used in Ahnesjö et al.^7^ sec. 2.4 and given here for the sake of overview.

Variable	Description
$c_{\theta }$	Fit parameter of the multiple‐scatter kernel describing the effective attenuation of the C‐term at transport direction θ (see eq. (15) in Ahnesjö et al. (2017)^7^)
$C_{\theta }$	Fit parameter of the multiple‐scatter kernel describing the magnitude of the C‐term at transport direction θ (see eq. (15) in Ahnesjö et al. (2017)^7^)
$f_{\theta }$	Fit parameter of the multiple‐scatter kernel describing the effective attenuation of the F‐term at transport direction θ (see eq. (15) in Ahnesjö et al. (2017)^7^)
$F_{\theta }$	Fit parameter of the multiple‐scatter kernel describing the magnitude of the F‐term at transport direction θ (see eq. (15) in Ahnesjö et al. (2017)^7^)
$\Delta \Omega_{m}$	Solid angle for the cone m of the energy deposition kernel
$S_{2\mathrm{sc}}$	Scerma (scatter energy released per mass) of second‐scatter photons (photons that have undergone one previous scattering) constituting the energy available for deposition of the multiple‐scatter dose $D_{\mathrm{msc}}$
$S_{2\mathrm{sc},j}$	The scerma partition released by second‐scatter photons to be transported along the line direction (see Fig. 1 in Ahnesjö et al. (2017)^7^)
$D_{\mathrm{msc}}$	Multiple‐scatter dose, dose deposited through interactions with photons which has been scattered at least twice after leaving the source and its encapsulation
$D_{\mathrm{msc},j}$	Multiple‐scatter dose partition absorbed at the jth step along the collapsed cone transport line
$\rho_{m}$	Mass density of the medium “m”
$\rho_{w}$	Mass density of water, the medium for which the energy deposition kernels are derived
$\rho_{j}$	Mass density in the iterated voxel
$\Delta l_{j}$	Length in the voxel of the jth step along the collapsed cone transport line (see Fig. 1 in Ahnesjö et al. (2017)^7^)
$\Delta l_{\max}$	Total step length per voxel for transport lines of the direction of interest
$\varepsilon_{j}$	$=\exp(-\eta_{\mathrm{msc},j}\cdot c_{\theta }\cdot \Delta l_{j})$
$\sigma_{j}$	$=S_{2\mathrm{sc},j}\rho_{j}\Delta \Omega_{m}\frac{C_{\theta }}{c_{\theta }}$
${\hat{R}}_{\mathrm{msc}C,j}$	Effective energy fluence of multiple‐scatter radiant energy for a transport line leaving the voxel at step j (cf. the text following eq. (50) in Ahnesjö et al. (2017)^7^)
$\Delta {\hat{R}}_{\mathrm{msc}C,j}$	Contribution to ${\hat{R}}_{\mathrm{msc}C,j}$ of multiple‐scatter from the voxel's scerma $S_{2\mathrm{sc},j}$ considering self‐absorption during step j
${\hat{R}}_{\mathrm{corr},j}$	Correction to $\Delta {\hat{R}}_{\mathrm{msc}C,j}$ in case ${\hat{R}}_{\mathrm{msc}C,j}$ is calculated discarding media specific deviations from water in $D_{\mathrm{msc},j}$ (i.e., neglecting χmsc,j/χmsc,jηmsc,jηmsc,j in Equation (2))
[μ¯en,msc/μ¯en,mscρρ]wm	Ratio for medium “m” to water of spectral averaged mass energy absorption coefficients for multiple‐scattered photons
[μ¯msc/μ¯mscρρ]wm	Ratio for medium “m” to water of spectral averaged mass attenuation coefficients for multiple‐scattered photons
$\chi_{\mathrm{msc},j}$	=ρmρw[μ¯en,msc/μ¯en,mscρρ]wm spectral averaged linear energy absorption coefficient ratio of multiple‐scattered photons for the voxel medium relative water at step j
$\eta_{\mathrm{msc},j}$	=ρmρw[μ¯msc/μ¯mscρρ]wm spectral averaged linear attenuation coefficient ratio of multiple‐scattered photons for the voxel medium relative water at step j

The msc kernel parameterization has two terms with proportionality factors C and F (see eq. (15) in Ahnesjö et al.^7^). For brevity, we show here only the updated formulas for the C‐term, keeping in mind that the F‐term must be similarly changed as well. Analogous to eq. (11) in Ahnesjö et al.^7^ for 1sc, we now introduce the factor χmsc/χmscηmscηmsc to correct the msc energy absorption for non‐water media, thus modifying eq. (21) in Ahnesjö et al.^7^ to instead follow as

(2) $$\begin{array}{ccc} D_{\mathrm{mscC},j} & = & \frac{1}{\Delta l_{\max}}\cdot \frac{1}{\rho_{j}}\frac{\chi_{\mathrm{msc},j}}{\eta_{\mathrm{msc},j}}\left[\left(1-\varepsilon_{j}\right)\cdot {\hat{R}}_{\mathrm{msc}C,j-1}\right.\\ & & +\left.\sigma_{j}\cdot \Delta l_{j}-\Delta {\hat{R}}_{\mathrm{msc}C,j}\right] \end{array}$$

for the dose deposition at the step *j* along a transport line for the C‐part of the msc dose. The different terms within the bracket of Equation (2) correspond to different processes during the step; the energy absorption from the fluence ${\hat{R}}_{\mathrm{msc}C,j-1}$ entering the voxel is proportional to $\left(1-\varepsilon_{j}\right)\cdot {\hat{R}}_{\mathrm{msc}C,j-1}$, the self‐absorption from $S_{2\mathrm{sc},j}$ neglecting radiative losses is proportional to $\sigma_{j}\cdot \Delta l_{j}$, and finally $\Delta {\hat{R}}_{\mathrm{msc}C,j}$ corrects for the radiative losses from the voxel contribution. The latter part adds to the transport to the next voxel along the line [c.f. eqs. (22) and (50) in Ahnesjö et al.^7^].

However, the local change in energy deposition induced by the introduction of χmsc/χmscηmscηmsc in Equation (2) imposes a distant effect since the change in local absorption must be balanced by a similar change in the energy transported for absorption elsewhere. This is fulfilled through a change in $\Delta {\hat{R}}_{\mathrm{msc}C,j}$ by a correction term ${\hat{R}}_{\mathrm{corr},j}$. If more energy is absorbed by the medium compared to that for water (as for, e.g., bone), less energy must leave the voxel as carried by ${\hat{R}}_{\mathrm{msc}C,j}$, and vice versa if less energy is absorbed. The term ${\hat{R}}_{\mathrm{corr},j}$ is determined from the difference in dose between the cases of including the absorption correction χmsc/χmscηmscηmsc compared to omitting it. The dose difference with a reversed sign and multiplied by $\Delta l_{\max}\rho_{j}$ (to convert from dose difference to effective energy fluence difference) thus yields

(3) $$\begin{array}{ccc} {\hat{R}}_{\mathrm{corr},j} & = & \left(1-\frac{\chi_{\mathrm{msc},j}}{\eta_{\mathrm{msc},j}}\right)\left[\left(1-\varepsilon_{j}\right)\cdot {\hat{R}}_{\mathrm{msc}C,j-1}\right.\\ & & +\left.\sigma_{j}\cdot \Delta l_{j}-\Delta {\hat{R}}_{\mathrm{msc}C,j}\right]. \end{array}$$

Introducing this difference into eq. (22) in Ahnesjö et al.^7^ gives finally

(4) $${\hat{R}}_{\mathrm{msc}C,j}={\hat{R}}_{\mathrm{msc}C,j-1}\cdot \varepsilon_{j}+\Delta {\hat{R}}_{\mathrm{msc}C,j}+{\hat{R}}_{\mathrm{corr},j}.$$

It follows directly that if χmsc,j/χmsc,jηmsc,jηmsc,j equals unity, as for water, ${\hat{R}}_{\mathrm{corr}}\to 0$ and Equation (4) becomes identical to eq. (22) in Ahnesjö et al.,^7^ as expected.

### 2.2 Phantoms

**FIGURE 1:**
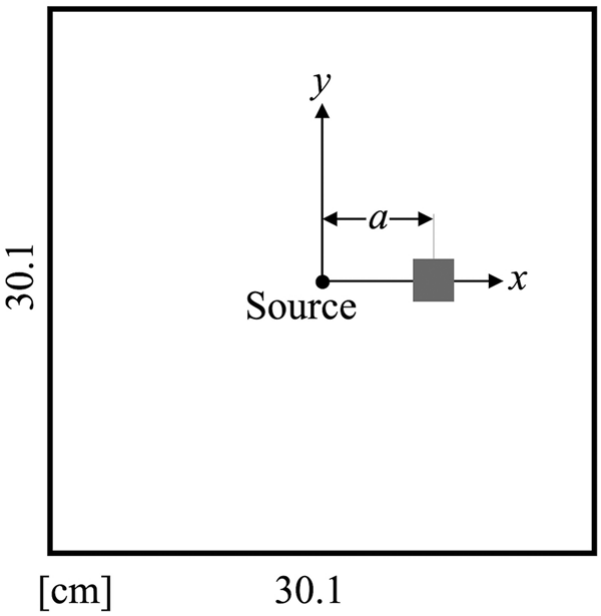
Outline through the center *xy*‐plane of the simulated 30.1 × 30.1 × 30.1 cm^3^ water phantom showing the location and dimensions of the box‐shaped heterogeneity. The Ir‐192 source is placed at the center and the bone/air heterogeneity is at distance *a* from the source, where *a* can be either 3.0 or 6.0 cm. The box is quadratic with a side of 2.1 cm in the *xy*‐plane and the length along the *z* direction is 3.1 cm.

The specifications for a generic ^192^Ir‐source given by the TG‐186 working group for MBDCA testing^19^ were used for dose calculations. The source was modeled at the center of a cubic water phantom of 30.1 cm side, see Figure 1. The source's longitudinal axis was aligned with the phantom's *z*‐axis. A boxed‐shaped heterogeneity of dimensions $2.1\;\times \;2.1\;\times \;3.1\;cm^{3}$, placed with its center at different distances from the source, yielded three test cases, two with the box filled with cortical bone (Case A, 6 cm from the source and Case B, 3 cm from the source) and one with the box filled with air (Case C, 3 cm from the source). For dose reporting (and dose scoring in the cases of MC), the phantom was divided into cubic voxels of size 0.1cm, and the dimensions and locations of the heterogeneities were chosen such that no voxel intersected any media boundary. The chemical compositions and densities of the media used for the phantom are presented in Table 2.

**TABLE 2:** Chemical composition of the phantom media.

Media	Chemical composition % by weight	Mass density g cm^−3^
Cortical bone^3^	H(3.4), C(15.5), N(4.2), O(43.5), Na(0.1), Mg(0.2), P(10.3), S(0.3), Ca(22.5)	1.92
Air^26^	N(75.5), O(23.2), Ar(1.3)	1.20E−03
Water^3^	H(11.2), O(88.8)	1.00

### 2.3 Dose calculations

Dose calculations were performed with a separate research software package based on the algorithm code in the Oncentra Brachy TPS. Dose distributions were calculated using the TG‐43 formalism, ACE, and ACE_corr_. All calculations with ACE and ACE_corr_ were performed in the high accuracy mode, which means 1620 and 240 transport directions for the 1sc and msc dose calculation, respectively,^7^ and a multiple resolution calculation grid, with cubic voxels of side 0.1 cm up to 10 cm from the source and coarser at larger distances. Details about the design of the multiple‐resolution grid can be found in Ahnesjö et al.^7^ After dose calculation, the multiple‐resolution dose grid was mapped by the software onto the dose reporting grid, and the results were compared to the dose from MC simulations. The MC simulations were performed with PENELOPE2018.^20^, ^21^ Configuration data used for the MC simulations are given in Table 3, as recommended by the RECORDS report.^22^ To enable separation by scatter generation in the same way as in ACE,^8^ specific dose scorers were implemented. These scorers consist of a set of rules to assign each particle with a bookkeeping number (bkn), using PENELOPE's ILB(5) label array. Primary photons are assigned bkn = 0. A primary photon is defined as a photon exiting through the source encapsulation, regardless of its interaction history inside the source or the source's encapsulation. However, for any particles re‐entering the source, the source is treated as part of the phantom. Any secondary charged particle (e.g., Compton electrons) keeps the bkn of its predecessor. A photon created in a secondary particle interaction (e.g., bremsstrahlung) or a photon interaction (such as Compton scattering) is given a bkn value of one higher than that of the interacting particle.

**TABLE 3:** Specifications of the Monte Carlo setup presented as recommended in the RECORDS^22^ report.

Item	Description	References
Code	(1) PENELOPE2018 (2) penEasy (v. 2019‐09‐21) Intel® Fortran compiler 18.0.3.	(1) Salvat^20^ (2) Sempau et al.^21^
Validation	Previously validated	Ballester et al.^19^
Timing	Average (sum of 120 parallel processes Intel® Xeon® Gold 6154 CPU @3.0 GHz): CPU time = 6700 h Number of histories = 1.9 × 10^12^	
Sources description	AAPM/ESTRO/ABG WG‐MBDCA, Generic HDR ^192^Ir source	Ballester et al.^19^
Cross‐sections	(1) Photoelectric: calculated with PHOTACS (2) Rayleigh scattering: using non‐relativistic perturbation theory (3) Compton: relativistic impulse approximation	(1) Sabbatucci et al.^27^ (2) Sakurai^28^, Born^29^, Baym^30^, Cullen et al.^31^ (3) Ribberfors^32^
Transport parameters	Photon cutoff = 1 keV. Electron transport disabled	
Variance reduction tools	Interaction forcing (Compton and photoelectric)	
Scored quantities	Collisional kerma (as surrogate of absorbed dose)	
History/Statistical uncertainties (*k* = 2)	Bone 6 cm: <0.8%, <0.4%, and <0.1% for *r* ≤ 10 cm, I ≤ 5 cm, and *r* ≤ 1 cm, respectively. Bone 3 cm: <0.7%, <0.3%, and <0.1% for *r* ≤ 10 cm, *r* ≤ 5 cm, and *r* ≤ 1 cm, respectively. Air: <0.9%, <0.9%, and <0.1% for *r* ≤ 10 cm, *r* ≤ 5 cm, and *r* ≤ 1 cm, respectively.	
Statistical method	History‐by‐history	Salvat^20^
Post processing	None	

## 3 RESULTS

**FIGURE 2:**
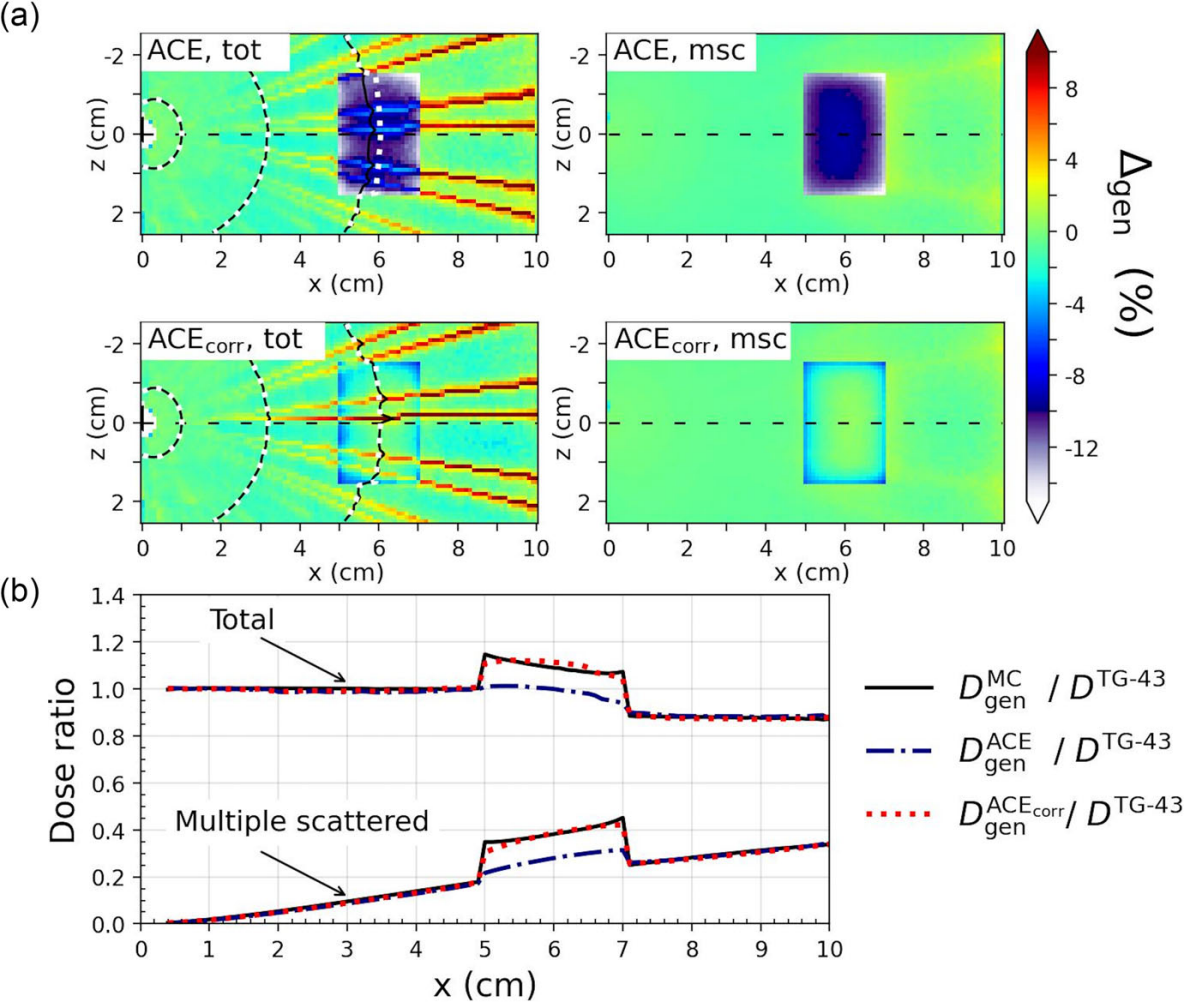
Comparison of ACE and ACE_corr_ to MC for Case A, with the cortical bone heterogeneity placed 6 cm from the source. (a) Percentage dose difference ratio ($\Delta_{\mathrm{gen}}$) maps of ACE (top two figures) and ACE_corr_ (bottom two figures) with MC as the reference for the total dose (left) and the msc dose (right). The 100%, 10%, and 3% isodose levels were calculated with MC (white dotted lines) and ACE/ACE_corr_ (black solid lines). (b) Dose profiles through the dashed black line in (a), that is, in the *y* = *z* = 0, plane for the total dose and the msc dose component, normalized to the dose calculated with TG‐43.

**FIGURE 3:**
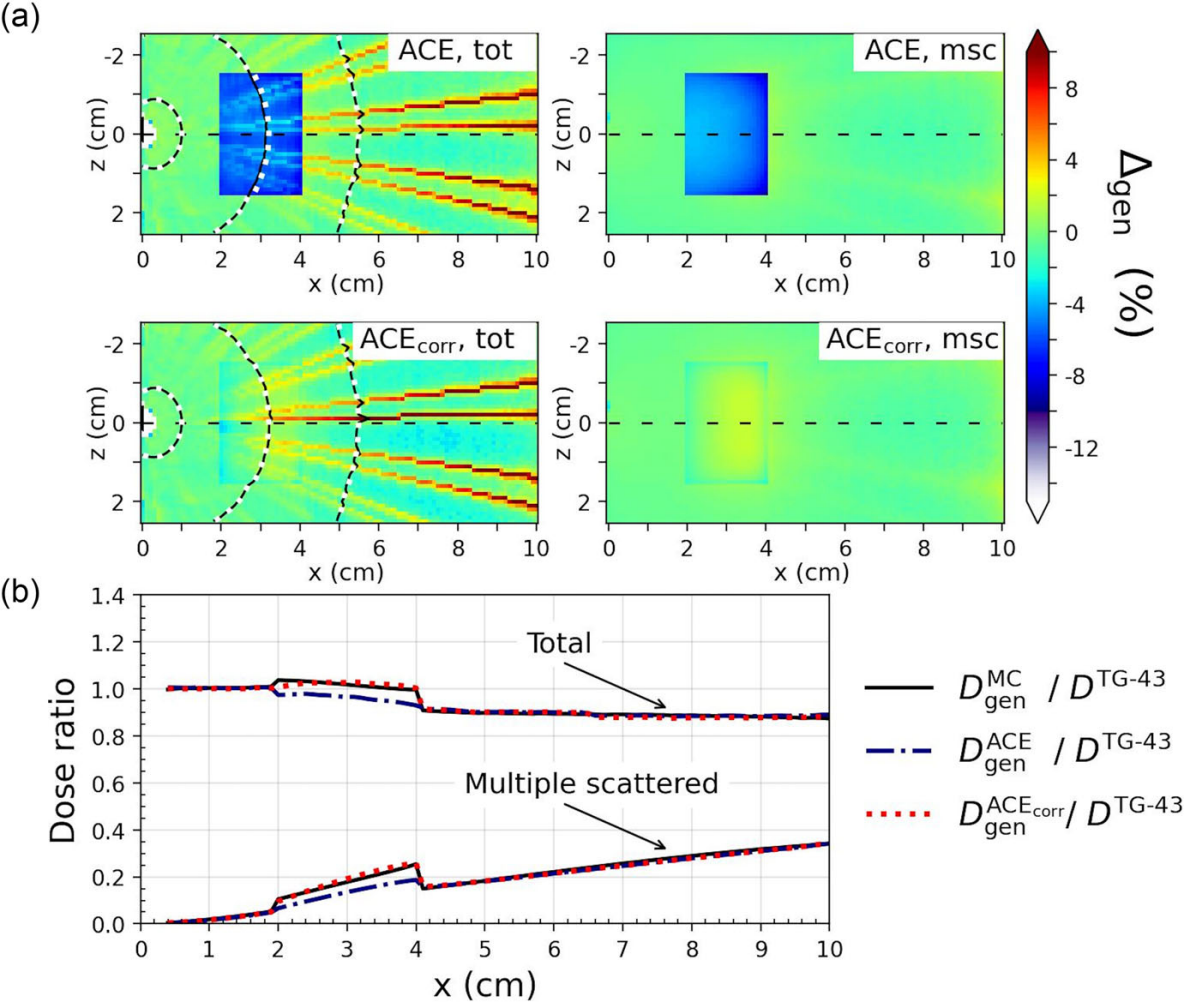
Comparison of ACE and ACEcorr to MC for Case B, with the cortical bone heterogeneity placed 3 cm from the source. (a) Percentage dose difference ratio ($\Delta_{\mathrm{gen}}$) maps of ACE (top two figures) and ACEcorr (bottom two figures) with MC as the reference for the total dose (left) and the msc dose (right). The 100%, 10%, and 3% isodose levels were calculated with MC (white dotted lines) and ACE/ACEcorr (black solid lines). (b) Dose profiles through the dashed black line in (a), that is, in the *y* = *z* = 0, plane for the total dose and the msc dose component, normalized to the dose calculated with TG‐43.

**FIGURE 4:**
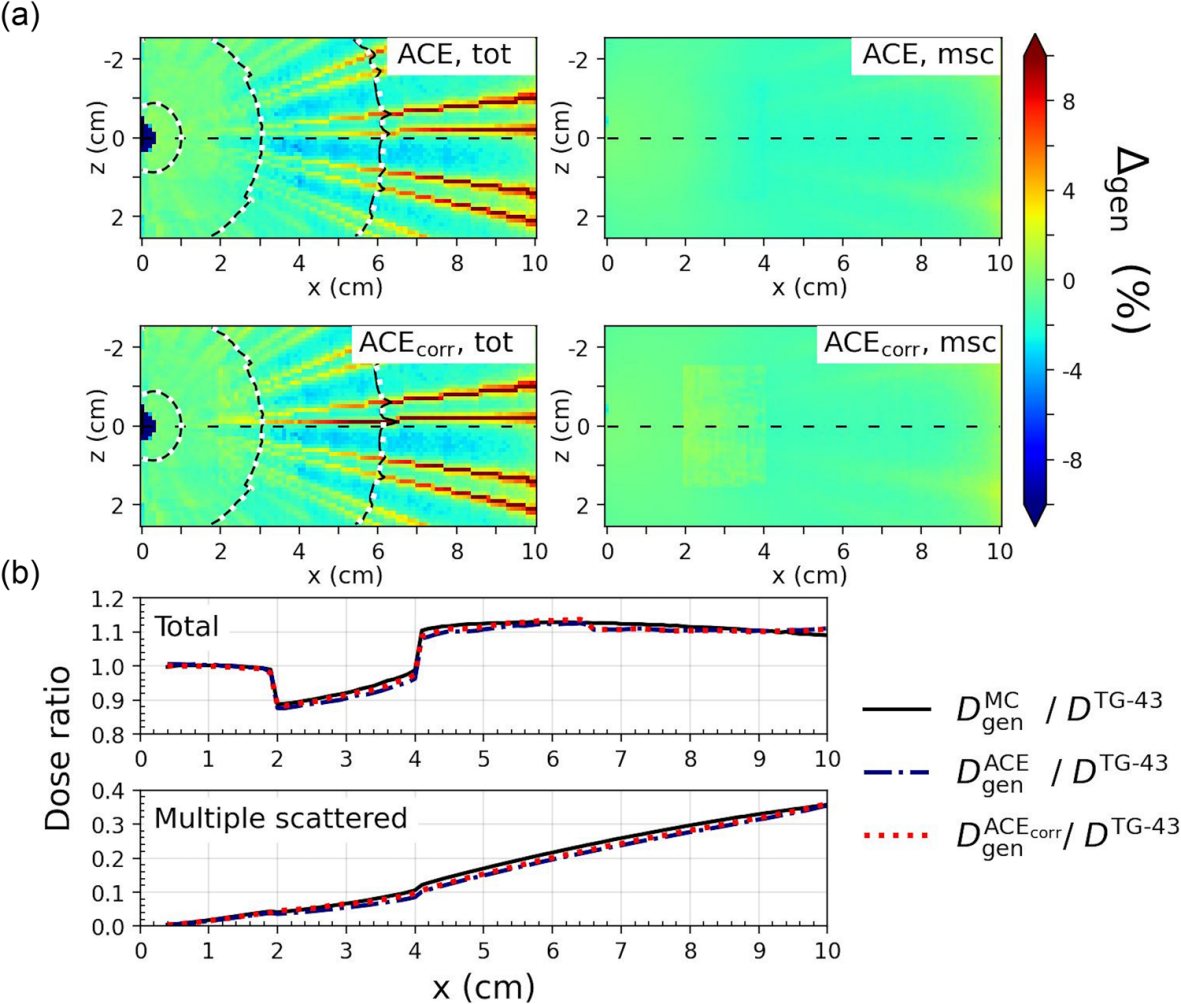
Comparison of ACE and ACE_corr_ to MC for Case C, with the air heterogeneity placed 3 cm from the source. (a) Percentage dose difference ratio ($\Delta_{\mathrm{gen}}$) maps of ACE (top two figures) and ACE_corr_ (bottom two figures) with MC as the reference for the total dose (left) and the msc dose (right). The 100%, 10%, and 3% isodose levels were calculated with MC (white dotted lines) and ACE/ACE_corr_ (black solid lines). (b) Dose profiles through the dashed black line in (a), that is, in the *y* = *z* = 0, plane for the total dose and the msc dose component, normalized to the dose calculated with TG‐43.

**FIGURE 5:**
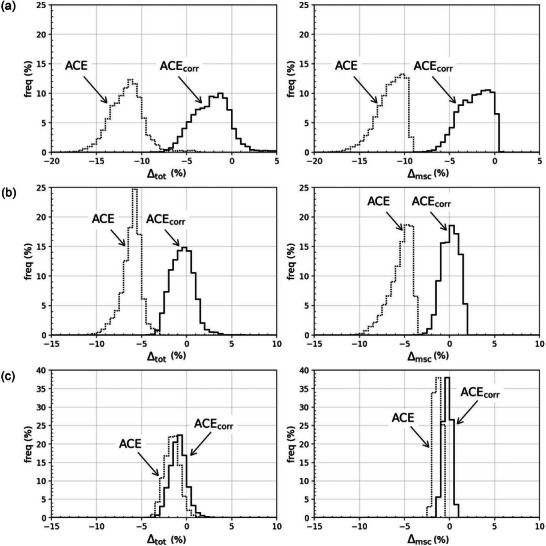
The distribution of $\Delta_{\mathrm{tot}}$ (left column) and $\Delta_{\mathrm{msc}}$ (right column) within the heterogeneities for Case A (top row), Case B (middle row), and Case C (bottom row). All histograms are calculated with a bin width of 0.5% and normalized to yield an integral value of 100. Statistical measures of the distributions can be found in Table 4.

The comparison of ACE and ACE_corr_ to MC are presented in Figures 2, 3, 4 for cases A–C, respectively, as percentage dose difference ratio ($\Delta_{\mathrm{gen}}$) maps and dose profiles for the total dose and the msc dose component. Table 3 presents the Type A maximum statistical uncertainties on $D_{\mathrm{tot}}^{\mathrm{MC}}$ at three different distances from the origin for each of the three cases. The distributions of $\Delta_{\mathrm{tot}}$ and $\Delta_{\mathrm{msc}}$ within the heterogeneities are presented in Figure 5, with the corresponding statistical measures given in Table 4. All distributions of $\Delta_{\mathrm{tot}}$, $\Delta_{\mathrm{prim}}$, and $\Delta_{\mathrm{msc}}$ had a skewness below, 1 whereas $\Delta_{1\mathrm{sc}}$ resulted in skewnesses up to 3.4. However, for all distributions, the difference between the mean (${\bar{\Delta }}_{\mathrm{gen}})$ and median values of $\Delta_{\mathrm{gen}}$ had a magnitude of ≤0.3%. The MC Type A uncertainties were well below 0.1% for all estimates of ${\bar{\Delta }}_{\mathrm{gen}}$ and the median values.

**TABLE 4:** The table presents statistical measures of the $\Delta_{\mathrm{gen}}$‐distributions in the heterogeneities for cases A–C.

		$\Delta_{\mathrm{tot}}$				$\Delta_{\mathrm{prim}}$				$\Delta_{1\mathrm{sc}}$				$\Delta_{\mathrm{msc}}$			
		${\bar{\Delta }}_{\mathrm{tot}}$	$\sigma_{\mathrm{tot}}$	Med.		${\bar{\Delta }}_{\mathrm{prim}}$	$\sigma_{\mathrm{prim}}$	Med.		${\bar{\Delta }}_{1\mathrm{sc}}$	$\sigma_{1\mathrm{sc}}$	Med.		${\bar{\Delta }}_{\mathrm{msc}}$	$\sigma_{\mathrm{msc}}$	Med.	
		(%)	(%)	(%)	Skew.	(%)	(%)	(%)	Skew.	(%)	(%)	(%)	Skew.	(%)	(%)	(%)	Skew.
A	ACE	−11.7	1.9	−11.6	0.3	0.0	0.1	0.0	0.1	−0.1	1.2	−0.3	2.7	−11.6	1.6	−11.4	−0.8
	ACE_corr_	−2.2	2.2	−2.2	0.7	0.2	0.1	0.2	0.0	−0.1	1.3	−0.4	3.4	−2.3	1.7	−2.1	−0.4
B	ACE	−6.1	1.1	−6.0	−0.7	−0.1	0.2	−0.1	−0.6	−0.6	0.6	−0.7	1.4	−5.5	1.3	−5.2	−1.0
	ACE_corr_	−0.5	1.2	−0.5	0.3	−0.1	0.1	−0.1	−0.3	−0.5	0.6	−0.6	1.8	0.1	0.9	0.1	−0.3
C	ACE	−1.6	0.9	−1.6	0.1	−0.1	0.3	−0.1	−0.1	−0.2	0.7	−0.3	1.5	−1.3	0.4	−1.3	0.0
	ACE_corr_	−0.9	0.9	−0.9	0.4	−0.6	0.3	−0.6	0.0	−0.1	0.7	−0.2	1.9	−0.3	0.5	−0.3	−0.1

The mean value (${\bar{\Delta }}_{\mathrm{gen}}$) is presented together with the standard deviation of the mean as a measure of the spread of $\Delta_{\mathrm{gen}}$‐values within the distribution. The table also presents the median (Med.) and skewness (Skew.) of the distributions. The MC type A uncertainties on all estimates of ${\bar{\Delta }}_{\mathrm{gen}}$ and the median values are below 0.1%. The comparison has been made for the three different scatter components (prim, 1sc, and msc), as well as for the total dose (tot), that is, the sum of the components. The distributions of $\Delta_{\mathrm{gen}}$ for the total and msc dose components are shown in Figure 5.

Large disagreements with ${\bar{\Delta }}_{\mathrm{tot}}$ = −11.7% between ACE and MC were observed within the cortical bone for Case A, with the cortical bone heterogeneity 6 cm from the source. Comparing the three dose components of ACE separately, ${\bar{\Delta }}_{\mathrm{gen}}$ for the primary and 1sc dose were within ±0.5% (${\bar{\Delta }}_{\mathrm{prim}}=\;0.0\%$ and ${\bar{\Delta }}_{1\mathrm{sc}}=\;-0.1\%$), whereas ${\bar{\Delta }}_{\mathrm{msc}}$ was −11.6%.

The updated algorithm ACE_corr_ showed better agreement with MC in the cortical bone for Case A, with ${\bar{\Delta }}_{\mathrm{tot}}=\;-2.2\%$. The improved accuracy of ACE_corr_ compared to ACE in the cortical bone was solely due to an improvement in the msc dose calculation, as ${\bar{\Delta }}_{\mathrm{msc}}\;=\;-\;2.3\%$ for ACE_corr,_ while the primary and 1sc dose components were only slightly affected by the updates (${\bar{\Delta }}_{\mathrm{prim}}=$0.2% and ${\bar{\Delta }}_{1\mathrm{sc}}=\;-0.1\%$). It can also be seen in Figure 5a that the histograms of $\Delta_{\mathrm{tot}}$ and $\Delta_{\mathrm{msc}}$ are centered closer to zero for ACE_corr_ than for ACE. The revised algorithm ACE_corr_ maintained the same calculation time as ACE.

ACE_corr_ was also superior to ACE for Case B, in which the cortical bone heterogeneity was located 3 cm from the source. This can be seen in the histograms of Figure 5b and by comparing the mean values of $\Delta_{\mathrm{gen}}$ in the heterogeneity between ACE and ACE_corr_. For ACE in the cortical bone heterogeneity ${\bar{\Delta }}_{\mathrm{msc}}$ was −5.5%, whereas for ACE_corr_ ${\bar{\Delta }}_{\mathrm{msc}}$ was 0.1%. The primary and 1sc dose calculation had only minor changes, ${\bar{\Delta }}_{\mathrm{prim}}=\;-0.1\%$ and ${\bar{\Delta }}_{1\mathrm{sc}}=\;-0.6\%$ for ACE and ${\bar{\Delta }}_{\mathrm{prim}}=\;-0.1\%$ and ${\bar{\Delta }}_{1\mathrm{sc}}=\;-0.5\%$ for ACE_corr_. Hence ACE_corr_ reduced the total dose underestimation in the cortical bone from ${\bar{\Delta }}_{\mathrm{tot}}=\;-6.1\%$ to ${\bar{\Delta }}_{\mathrm{tot}}=\;-0.5\%$.

In the low‐density air heterogeneity of Case C, the agreement between both ACE and ACE_corr_ to MC was within 2% (${\bar{\Delta }}_{\mathrm{tot}}=\;-1.6\%$ for ACE and ${\bar{\Delta }}_{\mathrm{tot}}=\;-0.9\%$ for ACE_corr_). The differences between ACE and ACE_corr_ were small and most visible for the msc dose component where ACE_corr_ was closer to MC (${\bar{\Delta }}_{\mathrm{msc}}=\;-$1.3% and ${\bar{\Delta }}_{\mathrm{msc}}=\;-0.3\%$ for ACE and ACE_corr_, respectively). The small improvement of ACE_corr_ compared to ACE is also visible in the histograms presented in Figure 5c.

## 4 DISCUSSION

The results showed that the underestimation made by ACE in cortical bone is indeed caused by the msc dose component. The corrected algorithm ACE_corr_ was superior to ACE for cortical bone heterogeneities, such as for Case A, where ${\bar{\Delta }}_{\mathrm{tot}}$ went from −11.7% for ACE to −2.2% for ACE_corr_. These results are significant compared to MC type A uncertainties. The largest effects of the updates were, as expected, found in the msc dose, although the updated data tables also caused a slight change in the primary and 1sc dose components. The larger (>1) and positive skewness found in the $\Delta_{1\mathrm{sc}}$‐distributions for both ACE and ACE_corr_ can be attributed to the discretization artifacts associated with the collapsed cone approximation, which are most pronounced in the first‐scatter dose distribution. Since the corrections to the algorithm only required minor code changes, there was no difference in calculation time between ACE and ACE_corr_. This can be expected to hold even in cases with larger heterogeneities as the algorithm calculation steps are the same regardless of voxel material.

As can be seen in Figures 2 and 3b, the ACE_corr_ radial dose profiles feature a more rounded shape through the bone inserts compared to MC. This results in underestimations at the interfaces of the cortical bone heterogeneities. The magnitude of these underestimations appears larger for Case A (≈6%) than for Case B (≈3%) due to the normalization to the total dose since there is a larger contribution of msc dose to the total dose in Case A. A possible reason for this phenomenon is a high absorption in cortical bone of the low energy components in the msc spectrum, modeled by MC but not by the bi‐exponential msc kernel used in ACE_corr_.

In addition to cortical bone, we tested the updates for an air heterogeneity. Although dose to air is of no clinical relevance, its correctness can serve as a marker of the algorithm's robustness to handle extreme conditions. Some discrepancies between ACE and MC in air have been noticed in earlier studies, for example, at patient boundaries.^13^ However, in this study, both ACE and ACE_corr_ agreed within 2% with MC ($|{\bar{\Delta }}_{\mathrm{msc}}|$< 2%). The closer agreement between ACE and MC seen in this study is likely due to the small size of the air cavity, which reduced the influence of the approximations in the msc dose calculation. Even though the differences between ACE and ACE_corr_ were small, a slight improvement for ACE_corr_ was still seen in the air heterogeneity.

To demonstrate the effects of the algorithm, we chose test cases of simple designs with shapes and sizes, exaggerated compared to clinical cases. With these simple test cases, the impact of the photoelectric effect on tissue materials was assessed. Hence, the study also demonstrates the importance of performing simple tests as, in complex cases, multiple algorithm behaviors can counteract each other, making the results difficult to interpret. However, before using the algorithm clinicaly, further tests should be performed, including the commissioning levels 1 and 2 described by TG‐186 and comparisons of ACE and ACE_corr_ for the clinical test Case provided by Peppa et al.^3^, ^23^, ^24^

The use of ACE_corr_ versus ACE mainly matters at lower dose levels since the relative contribution of the msc dose to the total dose increases with distance from the source. This can be seen by comparing cases A and B, where moving the heterogeneity 3 cm closer to the source reduced the magnitude of ${\bar{\Delta }}_{\mathrm{tot}}$ for ACE by approximately a factor of 0.5, from −11.7% to −6.1%. However, ACE did still underestimate the dose to the heterogeneity in case B, whereas ACE_corr_ was in much closer agreement to MC with ${\bar{\Delta }}_{\mathrm{tot}}=\;-0.5\%$. Furthermore, the positive effects of the update are expected to increase if the algorithm is used for lower energy sources, where the mass absorption and attenuation coefficients differ more from that of water for a larger number of tissue media.

It should also be mentioned that an alternative method to the heterogeneity scaling approach is to use media‐specific point kernels. Instead of having one kernel derived in water and scaled when used in non‐water media, this alternative method calls for the use of multiple kernels, one for each media. The media‐specific kernel approach is currently used in the modeling of shields of high atomic numbers.^7^, ^10^, ^25^ However, this option presents a significant drawback: it would require a large number of kernels to consider different media compositions for more variable body tissues.

## 5 CONCLUSIONS

The ACE algorithm is a commercially available MBDCA that has been shown to underestimate the dose in cortical bone.^13^, ^14^, ^15^, ^16^, ^18^ We have developed an algorithm update ACE_corr_ that introduces a mass absorption scaling factor into the multiple‐scatter dose calculation and also refined the derivation of interaction coefficients to account for spectral changes with distance from the source. We show that these updates improve the accuracy of dose to cortical bone with no change in calculation time. Due to increased generality, the updates are expected to improve the algorithm accuracy for a broad range of heterogeneous situations.

## CONFLICT OF INTERESTS STATEMENT

Bob van Veelen is currently employed by Elekta.

## References

[1] Nath R , Anderson LL , Luxton G , Weaver KA , Williamson JF , Meigooni AS . Dosimetry of interstitial brachytherapy sources: recommendations of the AAPM radiation therapy committee task group No. 43. American Association of Physicists in Medicine. Med Phys. 1995;22(2):209‐234. 10.1118/1.597458 7565352

[2] Perez‐Calatayud J , Ballester F , Das RK , et al. Dose calculation for photon‐emitting brachytherapy sources with average energy higher than 50 keV: report of the AAPM and ESTRO. Med Phys. 2012;39(5):2904‐2929. 10.1118/1.3703892 22559663

[3] Beaulieu L , Carlsson Tedgren Å , Carrier JF , et al. Report of the Task Group 186 on model‐based dose calculation methods in brachytherapy beyond the TG‐43 formalism: current status and recommendations for clinical implementation. Med Phys. 2012;39(10):6208‐6236. 10.1118/1.4747264 23039658

[4] Rivard MJ , Venselaar JLM , Beaulieu L . The evolution of brachytherapy treatment planning. Med Phys. 2009;36(6Part1):2136‐2153. 10.1118/1.3125136 19610303

[5] Enger SA , Vijande J , Rivard MJ . Model‐based dose calculation algorithms for brachytherapy dosimetry. Semin Radiat Oncol. 2020;30(1):77‐86. 10.1016/j.semradonc.2019.08.006 31727303

[6] Carlsson ÅK , Ahnesjö A . The collapsed cone superposition algorithm applied to scatter dose calculations in brachytherapy. Med Phys. 2000;27(10):2320‐2332. 10.1118/1.1290485 11099200

[7] Ahnesjö A , van Veelen B , Tedgren ÅC . Collapsed cone dose calculations for heterogeneous tissues in brachytherapy using primary and scatter separation source data. Comput Methods Programs Biomed. 2017;139:17‐29. 10.1016/j.cmpb.2016.10.022 28187887

[8] Carlsson ÅK , Ahnesjö A . Point kernels and superposition methods for scatter dose calculations in brachytherapy. Phys Med Biol. 2000;45(2):357. 10.1088/0031-9155/45/2/308 10701509

[9] Ahnesjö A . Collapsed cone convolution of radiant energy for photon dose calculation in heterogeneous media. Med Phys. 1989;16(4):577‐592. 10.1118/1.596360 2770632

[10] Carlsson Tedgren ÅK , Ahnesjö A . Accounting for high Z shields in brachytherapy using collapsed cone superposition for scatter dose calculation. Med Phys. 2003;30(8):2206‐2217. doi:high Z. 12945986 10.1118/1.1587411

[11] Carlsson Tedgren Å , Plamondon M , Beaulieu L . The collapsed cone algorithm for ^192^Ir dosimetry using phantom‐size adaptive multiple‐scatter point kernels. Phys Med Biol. 2015;60(13):5313‐5323. 10.1088/0031-9155/60/13/5313 26108232

[12] Carlsson Tedgren Å , Ahnesjö A . Optimization of the computational efficiency of a 3D, collapsed cone dose calculation algorithm for brachytherapy. Med Phys Lanc. 2008;35(4):1611‐1618. 10.1118/1.2889777 18491555

[13] Ma Y , Lacroix F , Lavallée MC , Beaulieu L . Validation of the oncentra brachy advanced collapsed cone engine for a commercial ^192^Ir source using heterogeneous geometries. Brachytherapy. 2015;14(6):939‐952. 10.1016/j.brachy.2015.08.003 26403533

[14] Duque AS , van Wagenberg T , Seidensticker M , et al. Validation of the collapsed cone algorithm for HDR liver brachytherapy against Monte Carlo simulations. Brachytherapy. 2021;20(4):936‐947. 10.1016/j.brachy.2021.03.018 34001415

[15] Terribilini D , Vitzthum V , Volken W , et al. Performance evaluation of a collapsed cone dose calculation algorithm for HDR Ir‐192 of APBI treatments. Med Phys. 2017;44(10):5475‐5485. 10.1002/mp.12490 28750134

[16] Peppa V , Pantelis E , Pappas E , Lahanas V , Loukas C , Papagiannis P . A user‐oriented procedure for the commissioning and quality assurance testing of treatment planning system dosimetry in high‐dose‐rate brachytherapy. Brachytherapy. 2016;15(2):252‐262. 10.1016/j.brachy.2015.11.001 26727331

[17] Abe K , Kadoya N , Sato S , et al. Impact of a commercially available model‐based dose calculation algorithm on treatment planning of high‐dose‐rate brachytherapy in patients with cervical cancer. J Radiat Res (Tokyo). 2018;59(2):198‐206. 10.1093/jrr/rrx081 29378024 PMC5951107

[18] Papagiannis P , Pantelis E , Karaiskos P . Current state of the art brachytherapy treatment planning dosimetry algorithms. Br J Radiol. 2014;87(1041):20140163. 10.1259/bjr.20140163 25027247 PMC4453149

[19] Ballester F , Carlsson Tedgren Å , Granero D , et al. A generic high‐dose rate ^192^Ir brachytherapy source for evaluation of model‐based dose calculations beyond the TG‐43 formalism. Med Phys. 2015;42(6Part1):3048‐3062. 10.1118/1.4921020 26127057

[20] Salvat F . Penelope‐2018: a code system for monte carlo simulation of electron and photon transport. Published online, 2019.

[21] Sempau J , Badal A , Brualla L . A PENELOPE‐based system for the automated Monte Carlo simulation of clinacs and voxelized geometries‐application to far‐from‐axis fields. Med Phys. 2011;38(11):5887‐5895. 10.1118/1.3643029 22047353

[22] Sechopoulos I , Rogers DWO , Bazalova‐Carter M , et al. RECORDS: improved reporting of montE CarlO RaDiation transport studies: report of the AAPM research committee task group 268. Med Phys. 2018;45(1):e1‐e5. 10.1002/mp.12702 29178605

[23] Peppa V , Thomson RM , Enger SA , et al. A MC‐based anthropomorphic test case for commissioning model‐based dose calculation in interstitial breast 192‐Ir HDR brachytherapy. Med Phys. 2023;50(7):4675‐4687. 10.1002/mp.16455 37194638

[24] Beaulieu L , Ballester F , Granero D , et al. AAPM WGDCAB Report 372: a joint AAPM, ESTRO, ABG, and ABS report on commissioning of model‐based dose calculation algorithms in brachytherapy. Med Phys. 2023;50(8):e946‐e960. 10.1002/mp.16571 37427750

[25] Ma Y , Vijande J , Ballester F , et al. A generic TG‐186 shielded applicator for commissioning model‐based dose calculation algorithms for high‐dose‐rate 192Ir brachytherapy. Med Phys. 2017;44(11):5961‐5976. 10.1002/mp.12459 28722180

[26] National Institute of Standards and Technology (NIST) . Composition of AIR, DRY (NEAR SEA LEVEL). Accessed March 4, 2024. https://physics.nist.gov/cgi‐bin/Star/compos.pl?matno=104

[27] Sabbatucci L , Salvat F . Theory and calculation of the atomic photoeffect. Radiat Phys Chem. 2016;121:122‐140. 10.1016/j.radphyschem.2015.10.021

[28] Sakurai JJ . Advanced Quantum Mechanics. 1st ed. Addison and Wesley; 1967.

[29] Born M . Atomic Physics. Blackie and Son; 1969.

[30] Baym G . Lectures on Quantum Mechanics. Westview Press; Published online 1974.

[31] Cullen DE , Hubbell JH , Kissel L . EPDL97: The Evaluated Photo Data Library 97 Version. 1997, 6. doi:10.2172/295438

[32] Ribberfors R . X‐ray incoherent scattering total cross sections and energy‐absorption cross sections by means of simple calculation routines. Phys Rev A. 1983;27(6):3061‐3070. 10.1103/PhysRevA.27.3061

